# Effect of replacement therapy (CRRT) and hemodialysis (IHD) on severe acute renal failure

**DOI:** 10.3389/fphar.2023.1122778

**Published:** 2023-07-21

**Authors:** Xiangyuan Zhang, Yinfang Yuan

**Affiliations:** ^1^ Critical Care Medicine, Shaoyang University Affiliated Second Hospital, Shaoyang, China; ^2^ Nephrology Department, Shaoyang University Affiliated Second Hospital, Shaoyang, China

**Keywords:** replacement therapy, hemodialysis treatment, severe acute renal failure, blood creatinine, ventilator therapy

## Abstract

Hyperkalemia, metabolic acidosis, and acute uremia are the main symptoms in patients with severe acute renal failure (SARF). Its clinical symptoms are obvious, and it is extremely harmful. It needs to take active and effective measures for treatment. CRRT refers to any extracorporeal blood purification treatment technique designed to replace impaired renal function for 24 h or nearly 24 h. Hemodialysis treatment is a treatment process in which the patient’s blood is discharged from the body, passes through the dialysis membrane and dialysis machine, removes excess toxins and water in the body, corrects electrolyte and acid-base disorders, and then returns the blood to the body. In order to explore the efficacy of replacement therapy and hemodialysis in the treatment of severe acute renal failure, the data samples were randomly divided into observation group and control group, who were given conventional treatment, hemodialysis and replacement therapy, respectively. Clinical data show that after replacement therapy and hemodialysis in patients with severe acute renal failure in the observation group, the levels of parathyroid hormone, renin, and quality of life were all improved, with an improvement rate of 9.47%, which has certain promotional value.

## 1 Introduction

Chronic renal insufficiency has evolved into one of the most dangerous conditions that endanger human health as a result of the rise in human living standards and the advancement of medical technology. Acute renal failure (ARF) refers to symptoms such as accumulation of nitrogenous wastes, water-electrolyte and acid-base imbalance caused by abnormal glomerular filtration rate. The renal function (glomerular filtration function) drops suddenly (within 48 h), which is manifested by an increase of absolute value of blood creatinine ≥ 0.3 mg/dl (≥26.5 μ Mol/l), or increase ≥ 50% (reaching 1.5 times of the baseline value), or urine volume<0.5 mL/(kg. h) for more than 6 h (discharging obstructive nephropathy or dehydration status). It not only causes great damage to kidney function, but also affects other systems of the body and seriously endangers the patient’s life. There isn't a perfect or efficient way of controlling at the moment. This article explores the effect of replacement therapy (CRRT) and hemodialysis (IHD) in the clinical treatment of patients with severe acute renal failure.

There are scholars related research on hemodialysis. Through research, Kistler B M found that hemodialysis, as a conventional treatment for blood purification, could effectively remove excess water and nitrogen waste in patients to achieve acid-base and water-electrolyte balance (Kistler et al., 2018),Hemodialysis has two main advantages. First, it can remove toxins in a short time and stabilize the internal environment of the body. Second, it is easier to get medical treatment. Castillo-Torres S A believed that the rapid clearance rate could negatively affect the patient’s hemodynamics. It caused complications such as electrolyte imbalance and hypotension, which made the efficacy poor (Castillo-Torres et al., 2018). Wanner believed that patients on intermittent hemodialysis should receive intermittent hemodialysis once a week. Compared with intermittent hemodialysis, continuous hemodialysis was hemodynamically stable. It was easy and unrestricted to adequately rehydrate, which provided better metabolic control and clearance of the body’s inflammatory mediators (Kirsch et al., 2017). Lin S Y found that most scholars believed that continuous blood purification (CBP) was safe and well tolerated in patients with severe ARF, which was especially suitable for patients who were prone to hypotension and cardiac insufficiency during IHD treatment (Lin and Yi-Wen, 2018), If the patient’s blood pressure is low, it may aggravate hypotension, cardiac insufficiency or arrhythmia during blood purification. Block G A believed that compared with IHD, CBP removed water and solutes through an isotonic method and continuously adjusted the fluid balance to remove more fluid, which was more in line with physiological conditions (Block et al., 2017). Sabatino proposed that isotonic ultrafiltration facilitated plasma refilling and stabilized the renin-angiotensin system. The body’s response to vasoactive substances was enhanced, normal organ perfusion was maintained. Extracellular fluid osmotic pressure was stabilized, and body temperature was dropped slightly during treatment. It maintained hemodynamics better, which was beneficial to the recovery of organ function such as kidneys (Sabatino et al., 2017). Shafi T believed that when CBP was used for the treatment of severe ARF, the treatment mode, the composition of replacement fluid and dialysate were also important factors that affected the treatment effect. No matter what treatment method was adopted, large fluctuations in blood pH should be avoided (Shafi et al., 2017), It will affect all kinds of metabolism of the human body, as well as abnormal physiological functions. IHD has the ability to remove solutes with high efficiency. The basic principle of IHD treatment is slow, continuous removal of solutes and pathogenic mediators to maintain a stable internal environment, which makes it more physiological and avoids adverse effects on other organs.

There are now scholars related research on alternative therapy. Wang C research found that replacement therapy (CRRT) was a new method of blood purification treatment. It mainly removed excess water and nitrogen-containing wastes in the patient’s body through a slow isotonic method, and adjusted the fluid balance in the body according to the actual needs of the patient, which was more consistent with how the human body functions physiologically (Wang et al., 2017), It can make the patient’s internal and external circulation smooth and avoid blood congestion. Yang X M believed that this method could effectively ensure the clearing effect after long-term continuous treatment. At the same time, it could enhance the immune regulation ability of patients and meet their nutritional needs (Yang et al., 2017). Robinski found through research that renal replacement therapy mostly used puncture technology to build a vascular circuit in the patient’s body. Renal replacement therapy was a crucial component of the care for patients with acute kidney damage, particularly those who were severely sick (Robinski et al., 2017). Bagshaw S M suggested that renal replacement therapy (RRT) was not just routine supportive care for critically ill patients. The emerging technology of continuous renal replacement therapy was still the focus of attention (Bagshaw and Wald, 2017), Continuous renal replacement therapy is one of the most commonly used blood purification technologies in the rescue of critically ill patients, which mimics the filtration principle of the glomerulus. Lumlertgul N believed that acute renal failure referred to a groups of common clinical primary or secondary renal damage, especially in critically ill patients such as multiple organ dysfunction syndrome and severe sepsis. Such as blood urea nitrogen, serum creatinine (Scr), they are also affected by various factors such as age, race, metabolic status, volume status, muscle mass, medications, and infections. Especially when the infection is severe, the rate of Scr production is reduced (Lumlertgul et al., 2018). For critically ill patients and patients complicated with ARF after surgery, early initiation of dialysis can achieve a higher survival rate. Delmas C study found that renal replacement therapy in critically ill patients required not only the elimination of metabolic toxins to maintain the acid-base balance of water, electrolytes, but also supportive treatment of patients to eliminate inflammatory mediators in the body and ensure the supply of antibiotics, nutrients and other fluids. This promotes renal recovery and avoids further complications of ARF (Delmas et al., 2018). With the development and progress of renal replacement therapy, the prognosis of patients with severe acute renal failure has improved significantly. However, there are still great controversies about the dosage, choice of treatment methods and timing of treatment for replacement therapy (Lv and Qiao, 2020; Xie et al., 2021).

Severe acute renal failure is caused by chronic kidney damage caused by primary glomerulonephritis, diabetes and hypertension, which in turn causes substantial damage to the kidneys. It shrinks the kidneys significantly and cannot maintain basic functions. Different age, type of etiology and severity of patients with severe renal failure have a great impact on replacement therapy and hemodialysis treatment (Ayad et al., 2021). More consideration should be given to the available treatments in light of the unique circumstances faced by individuals with severe acute renal failure. The clinical impact of hemodialysis and alternative treatment in individuals with severe acute renal failure has been discussed in this research.

## 2 Cause of severe acute renal failure

**FIGURE 1:**
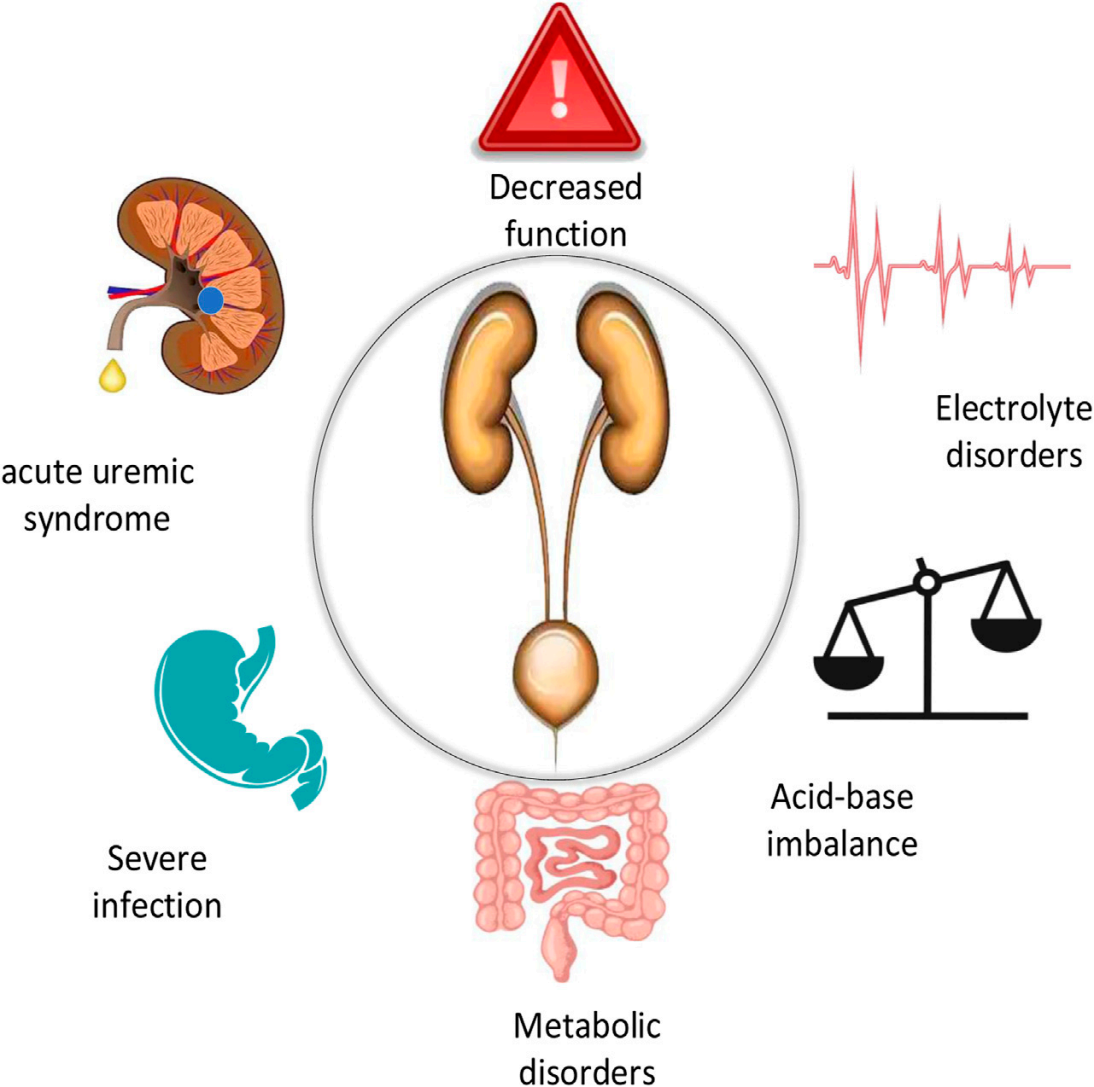
Damage caused by acute renal failure.

**FIGURE 2:**
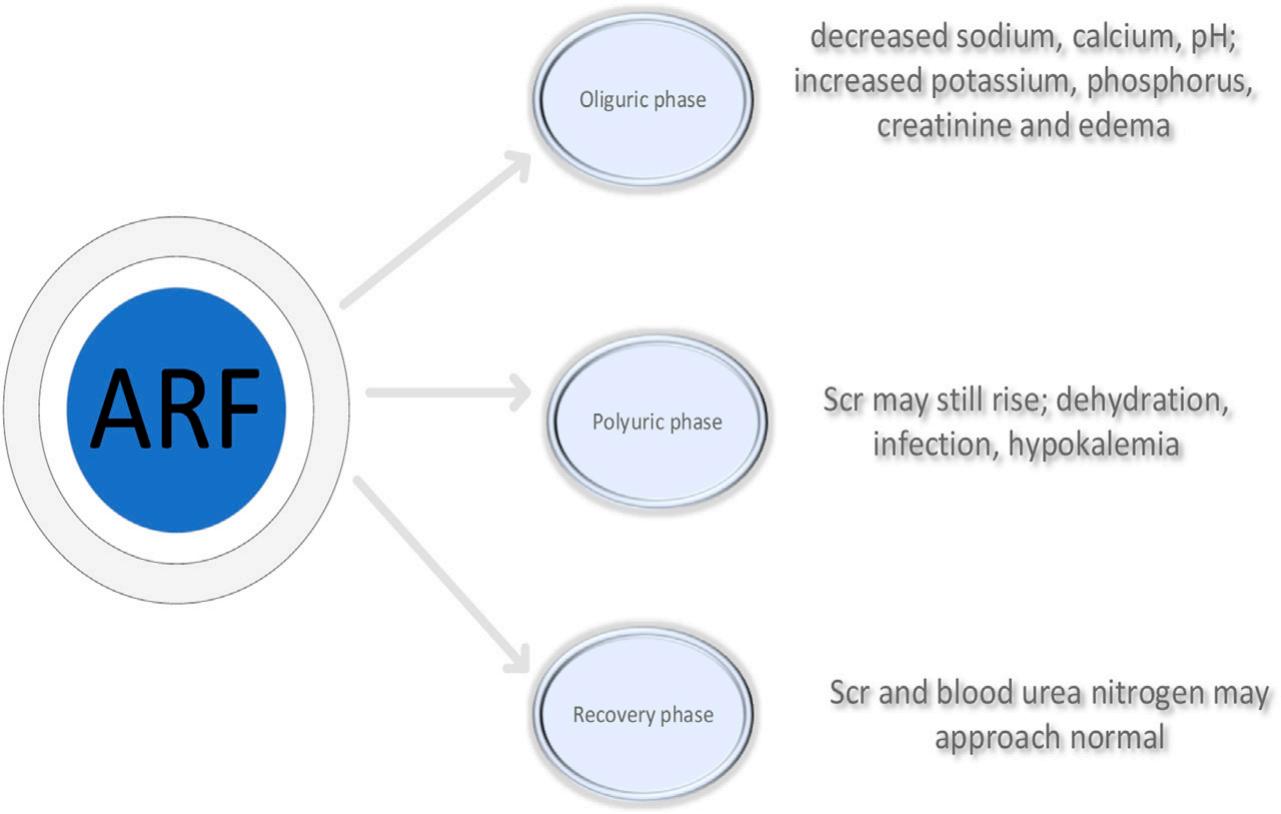
Three stages of acute renal failure: oliguria, polyuria and recovery.

A prevalent clinical acute condition is acute renal failure (ARF). The renal function damage caused by various reasons causes the patient’s corresponding adjustment function to drop sharply within a few days or even hours, resulting in water and electrolyte disturbances, acid-base imbalances and metabolic disorders. It may also lead to a series of complications such as acute uremia, hyperkalemia and metabolic acidosis, most of which are secondary to complicated surgery, severe infection and poisoning (Chen and Chen, 2019), as shown in Figure 1. The healing process of ARF has three periods: oliguria, polyuria and recovery, as shown in Figure 2. The clinical manifestations of oliguria are mainly the decrease of sodium, calcium and pH value, the increase of potassium, phosphorus and creatinine, and the symptoms of edema. Some patients with uremia may be accompanied by symptoms such as nausea, vomiting, gastrointestinal bleeding, heart failure, and dyspnea (Zin and Akula, 2021), The blood system, endocrine system and nervous system are seriously involved. In the early stage of polyuria, although the urine volume increases, the glomerular filtration rate is still very low, so the serum creatinine and urea nitrogen can still be significantly increased, and the symptoms of metabolic acidosis and uremia can still be serious. During the recovery period, Scr and blood urea nitrogen can be close to normal values, and urine output can gradually return to normal. After further effective treatment, the glomerular filtration function can be recovered after 3–12 months of age (Lischkova, 2018). Patients usually have a poor prognosis, such as hemodynamic instability, volume overload, etc (Srinivasa et al., 2018). Significant advancements in the diagnosis and treatment of ARF have been made recently. However, the spectrum of its primary disease has also undergone major changes, from simple ARF to acute, complex and more severe SARF (severe acute renal failure) (Hawkins et al., 2018).

**FIGURE 3:**
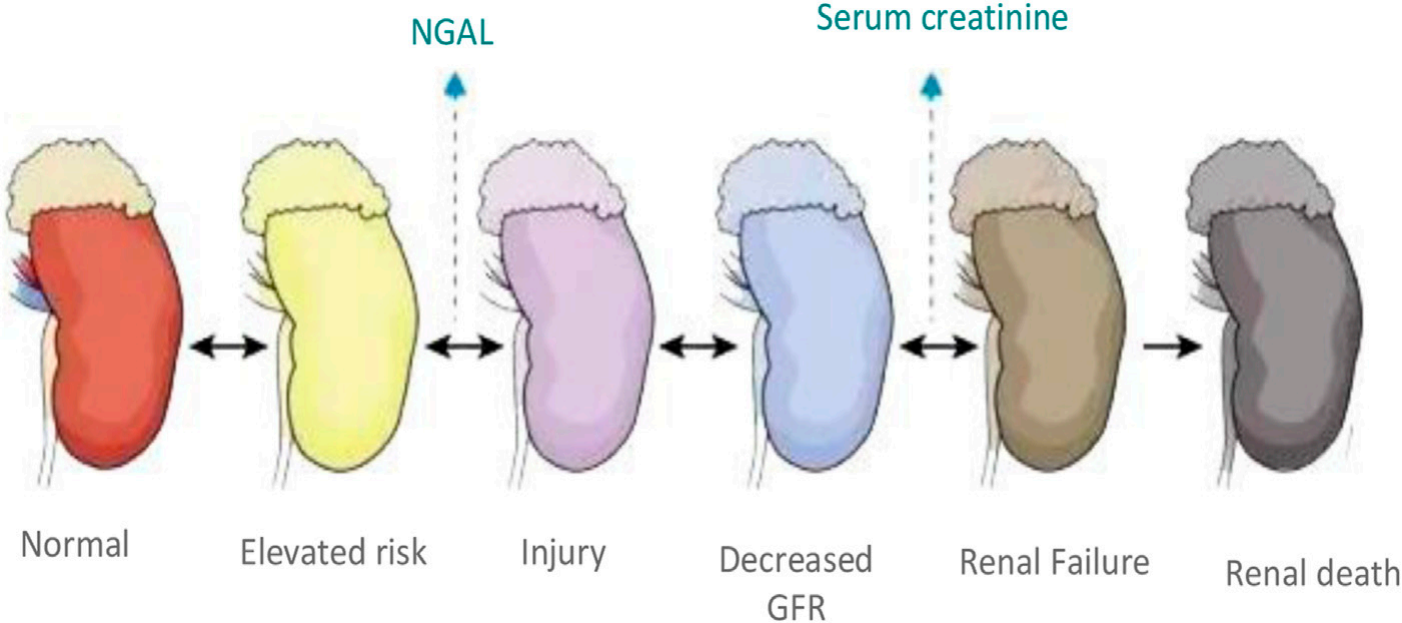
Progression of severe acute kidney injury.

Severe acute renal failure manifests as unstable cardiovascular function or cerebral edema, hypercatabolism, multiple organ dysfunction syndrome, etc. Patients with severe acute renal failure have significant pathophysiological changes such as metabolic toxicity, cytokine hypersecretion, accumulation of urea nitrogen and other metabolites, and a series of complications (Xie and Chen, 2018). Due to severe infections or simultaneous heart and brain organ failure, patients with severe acute renal failure often die, as shown in Figure 3. Although there has been a lot of study on the pathophysiology and therapy in recent years, the prognosis is still not the best. It is possible to gradually progress to chronic renal failure, that is, the fifth stage of chronic kidney disease, uremic stage. According to statistics, the incidence of severe acute renal failure is high. Death rate can reach approximately 47.2%. Early diagnosis and timely rescue are essential to reduce mortality, improve prognosis and restore renal function. Due to the wide variety of pathogenic factors of severe acute renal failure, the clinical manifestations of different patients are different, the clinical diagnosis and treatment plan are difficult to determine, and the prognosis of patients with different treatment plans is different (Cikova et al., 2017). On the basis of common treatments such as correction of water-electrolyte and acid-base balance disorders, anti-infection, nutritional support, etc., specific treatment plans and strengths need to be determined according to the etiology, combined with the diagnosis and the nature of the disease.

It is a difficult problem for doctors to identify the cause of severe acute renal failure and take corresponding treatment. This is mainly because the symptoms of diseases of different causes are similar, but there are essential differences in the treatment process. Sometimes if not handled properly, the condition would worsen more rapidly.

Severe acute renal failure is more severe from a clinical perspective. It is unusual and treatable from the standpoint of the body’s primary organs. Therefore, a thorough understanding of the clinical symptoms and causes of death in severe acute renal failure is critical to reducing the morbidity and mortality of severe acute renal failure. In order to raise the rate of illness diagnosis for increasing the efficacy of therapy and decreasing the rate of mortality, and enhance patient quality of life, it is required to investigate, evaluate, and debate the etiology, treatment, and regression.

## 3 Experiments on replacement therapy (CRRT) and hemodialysis (IHD)

### 3.1 Materials and methods

Eighty instances of severe acute renal failure were gathered and examined in this research. All 80 cases of severe acute renal failure met the criteria. Both groups have reached the prescribed diagnostic criteria for severe acute renal failure, and all cases have been approved. Cardiovascular, cerebrovascular disease and cancer patients and those prone to drug allergic reactions have been excluded.

**FIGURE 4:**
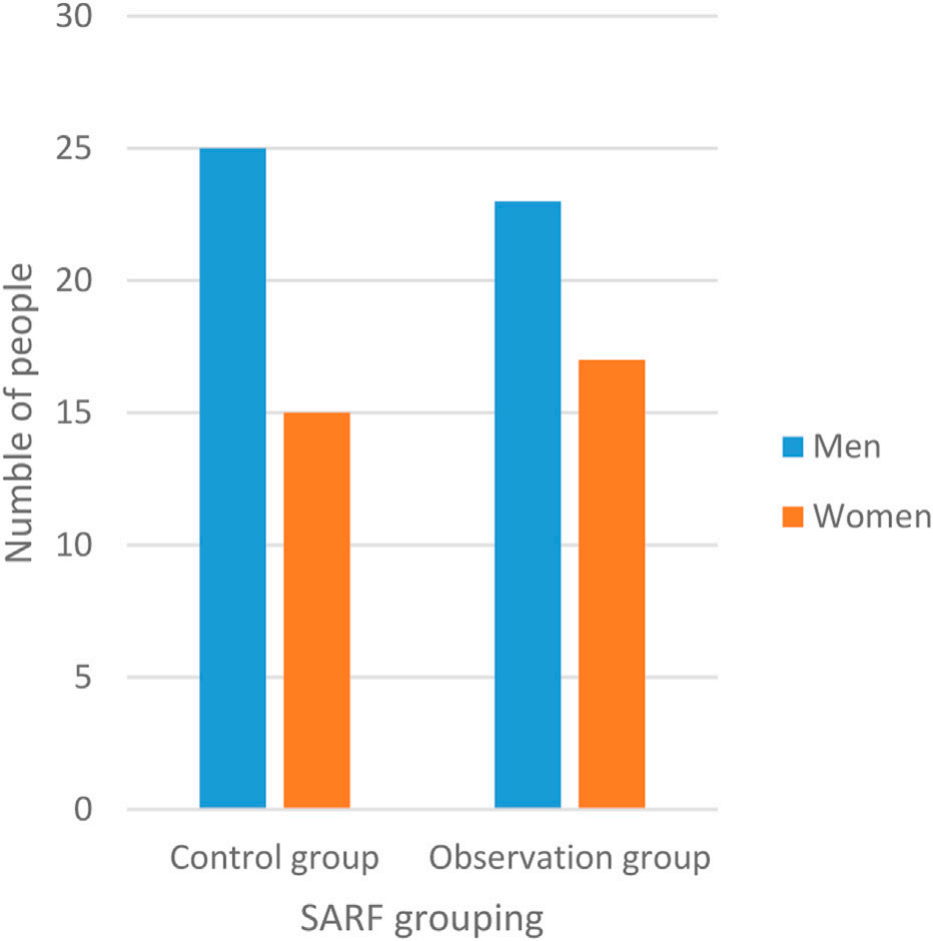
Personnel in the observation and control groups in SARF cases.

In this study, 80 severe acute renal failure patients were divided into two groups, each with 40 cases: the observation groups of severe acute renal failure patients and the control groups of severe acute renal failure patients. The observation groups consisted of 40 patients, with 23 men and 17 women suffering from severe acute renal failure. The youngest patient was 55 years old, and the oldest was 75 years old. The average age of the patients was approximately (65 ± 2.10) years. The control groups, on the other hand, consisted of 25 men and 15 women suffering from severe acute renal failure. Among the 40 patients, the oldest patient was 75 years old, (*p* > 0.05), which was comparable. Figure 4.

### 3.2 Experimental studies of treatment methods

The control groups was treated with bicarbonate dialysate for hemodialysis. The dialysate flow rate was 500 mL/min, the blood flow rate was 200–250 mL/min.The dialysis times were 2–3 times/week, and each dialysis time was 4–4.5 h. The observation groups performed renal replacement therapy by establishing vascular access for the patients, usually in the subclavian and internal jugular veins. Initially, the dose of low molecular weight heparin was 0.3–0.5 mg, and the dose was increased according to the patient’s recovery, usually 2–10 mg/L. The blood flow was 250 mL per minute and the treatment time was 10–12 h.

### 3.3 Statistical methods

Statistical analysis software was used to organize relevant data. Data were shown as positive correlation, and the relationship between data was expressed as standard deviation (X ± S). The chi-square test was first performed on the measured experimental data. Then the *t*-test was performed on the experimental data that met the conditions in which the differences were assumed to be statistically significant for comparison.

## 4 Statistics based on *t*-test

### 4.1 T-test statistics

When comparing two normal population means with equal variances, the *t*-test is used to determine whether the difference is statistically significant. Analysis of variance is used to determine the significance of discrepancies between several normal population means.

For the convenience of description, some notation is introduced. It is noted that $A_{1},A_{2},A_{3},\cdots ,A_{k}$ is the k different levels of factor A, X is the observation $X_{i1},X_{i2},X_{i3},\cdots ,X_{in_{i}}$ of the $n_{i}$ observations of indicator X at the $A_{i}$ level, $X_{i1},X_{i2},X_{i3},\cdots ,X_{in_{i}}∼N\left(\mu_{i},\sigma^{2}\right)$ ,

$${\bar{X}}_{i}=\frac{i}{n_{i}}\sum_{j=1}^{n_{i}}X_{ij}$$ (1)

$$S_{i}^{2}=\frac{1}{n_{i}-1}{\sum_{j=1}^{n_{i}}\left(X_{ij}-\bar{X_{i}}\right)}^{2}$$ (2)

$$n=\sum_{i=1}^{k}n_{i}i=1,2,\cdots ,k$$ (3)

$${\bar{X}}_{i}=\frac{i}{n_{i}}\sum_{i=1}^{k}\sum_{j=1}^{{n_{i}}_{i}}X_{ij}$$ (4)

$$SARF=\sum_{i=1}^{k}n_{i}{\left(X_{i}-\bar{X}\right)}^{2}$$ (5)

$$SARF=\sum_{i=1}^{k}\sum_{j=1}^{n_{i}}{\left(X_{i}-\bar{X}\right)}^{2}$$ (6)

The quantile $t_{a/2}\left(n\right)$ satisfies:

$$P\left(\left|t\right|\geq ta/2\left(n\right)\right)=a$$ (7)

The quantile $F_{a}\left(m,n\right)$ satisfies:

$$P\left(F\geq F_{a}\left(m,n\right)\right)=a$$ (8)

In the test hypothesis, when $H_{0}:\mu_{1}=\mu_{2},VS\;H_{0}:\mu_{1}\neq \mu_{2},$ , the t test and the two-level ANOVA are two equivalence tests. The reason for this is that the test statistic for this t -test is:

t=X1¯−X2¯Sw1n1−1n2 (9)

Among them, $n=n_{1}+n_{2}$ ,

$$S_{w}^{2}=\frac{\left(n_{1}-1\right)S_{1}^{2}+\left(n_{2}-1\right)S_{2}^{2}}{n-2}$$ (10)

When the null hypothesis $H_{0}$ holds, $t∼t\left(n-2\right)$ .

t2=X1¯−X2¯2Sw21n1−1n2=n1n2n1+n2X1¯−X2¯2Sw2∼F1,n−2 (11)

For two-level ANOVA, $\bar{X}$ can be deformed to $\frac{n_{1}X_{1}+n_{2}X_{2}}{n_{1}+n_{2}}$ :

$$\left.SARF=\sum_{i=1}^{2}n_{i}{\left(\bar{X_{1}}-\bar{X}\right)}^{2}=n_{1}{\left(\bar{X_{1}}-\bar{X}\right)}^{2}+n_{2}{\left(\bar{X_{2}}-\bar{X}\right)}^{2}\right)$$ (12)

$$IHD=n_{1}\left(\bar{X_{1}}-\frac{n_{1}\bar{X_{1}}-n_{2}\bar{X_{2}}}{n_{1}+n_{2}}\right)+n_{2}\left(\bar{X_{2}}-\frac{n_{1}\bar{X_{1}}-n_{2}\bar{X_{2}}}{n_{1}+n_{2}}\right)$$ (13)

$$CRRF=\frac{n_{1}n_{2}^{2}{\left(\bar{X_{1}}-\bar{X_{2}}\right)}^{2}}{{\left(n_{1}+n_{2}\right)}^{2}}+\frac{n_{1}^{2}n_{2}{\left(\bar{X_{1}}-\bar{X_{2}}\right)}^{2}}{{\left(n_{1}+n_{2}\right)}^{2}}$$ (14)

$$CRRF=\frac{n_{1}n_{2}}{n_{1}+n_{2}}{\left(\bar{X_{1}}-\bar{X_{2}}\right)}^{2}$$ (15)

$$IHD=\frac{CRRF}{n-2}=\frac{\left(n_{1}-1\right)S_{1}^{2}+\left(n_{2}-1\right)S_{2}^{2}}{n-2}$$ (16)

$$\frac{\left(n_{1}-1\right)S_{1}^{2}+\left(n_{2}-1\right)S_{2}^{2}}{n-2}=S_{w}^{2}$$ (17)

The test statistic is:

$$F=\frac{CRRF}{IHD}\approx \frac{n_{1}n_{2}}{n_{1}+n_{2}}\frac{{\left(\bar{X_{1}}-\bar{X_{2}}\right)}^{2}}{S_{w}^{2}}=t^{2}$$ (18)

$$P\left(\left|t\right|\geq ta/2\left(n-2\right)\right)=P\left(t^{2}\geq t_{a/2}^{2}\left(n-2\right)\right)$$ (19)

$$P\left(F\geq t_{a/2}^{2}\left(n-2\right)\right)=P\left(F\geq F_{a}\left(1,n-2\right)\right)=a$$ (20)

Therefore, when testing hypothesis $H_{0}:\mu_{1}=\mu_{2},VS\;H_{0}:\mu_{1}\neq \mu_{2}$ , the t test and the variance of the two levels are equivalent, and there is:

$$t_{a/2}^{2}\left(n-2\right)=F_{a}\left(1,n-2\right)$$ (21)

### 4.2 Observation of indicators

#### 4.2.1 Comparison of calcium and phosphorus metabolism between the two groups

The calcium and phosphorus metabolism in the observation groups was obviously due to the control groups, according to Table 1. The serum phosphorus and serum calcium of the control groups were 2.14 ± 0.32 and 2.75 ± 0.66, respectively, and the T value was 4.214. The serum phosphorus and serum calcium of the observation groups were 1.66 ± 0.14 and 1.81 ± 0.55, respectively, and the T value was 4.920. The difference was statistically significant (*p* < 0.05).

**TABLE 1:** Comparison of calcium and phosphorus metabolism between the two groups.

Groups	Observation groups	Control groups
Serum phosphorus	1.66 ± 0.14	2.14 ± 0.32
Serum calcium	1.81 ± 0.55	2.75 ± 0.66
T-value	4.920	4.214
*p*-value	<0.05	<0.05

#### 4.2.2 Observation of the levels of parathyroid hormone and renin each of the two groupings

The parathyroid hormone level of patients with severe acute renal failure in the observation groups was (46.6 ± 5.8) pg/L. The renin level of patients in the observation groups was (0.6 ± 0.12) pg/L. The parathyroid hormone level of patients with severe acute renal failure in the control groups was (88.5 ± 6.7) pg/L, and the renin level of patients with severe acute renal failure in the control groups. The renin level of the patients was (1.23 ± 0.22) ug/L.h. Thus, the observation groups with severe acute renal failure had a higher parathyroid hormone level than the control groups. The differences in the experimental data comparison were statistically significant, as shown in Table 2.

**TABLE 2:** Comparison of the two groups in terms of quality of survival.

Groups	Physical health	Mental health	Social relationship	Environmental factors	Parathyroid hormone	Renin levels
Observation groups (*n* = 40)	8.61 ± 10.2	80.1 ± 9.2	87.3 ± 9.7	88.5 ± 11.6	46.6 ± 5.8	0.6 ± 0.12
Control groups (*n* = 40)	63.1 ± 8.7	52.3 ± 10.6	65.9 ± 9.8	70.2 ± 10.3	88.5 ± 6.7	1.23 ± 0.22
T-value	3.956	3.659	3.562	3.3325	3.127	3.012
*p*-value	<0.05	<0.05	<0.05	<0.05	<0.05	<0.05

Comparison of the quality of survival status between the observation and control groups.

When observing the survival quality indicators of patients with severe acute renal failure in the control groups and the observation groups, it was found by observing the experimental data that the clinical effect of patients with severe acute renal failure in the observation groups was as high as about 90%. While the clinical effect of patients with severe acute renal failure in the control groups was only 72.5%. Therefore, it was concluded that the survival quality indexes of patients with severe acute renal failure in the observation groups were significantly better than those in the control groups, as shown in Table 3.

**TABLE 3:** Comparison of clinical results between two groups of patients.

Groups	n	Apparent outcome	Effective	Ineffective	Total effective rate (%)
Treatment groups	40 cases	31	5	4	90
Control groups	40 cases	18	11	11	72.5

The improvement of renal function and cytokine indices were examined before and after administering various treatment regimens. The findings demonstrated that the control groups was responsible for the experimental group’s improved SCR, BUN, and Hs-CRP factor levels. After treatment, the observation groups’s SCR, BUN, and Hs-CRP factors were 301 ± 47.3 (mol/L), 38.1 ± 2.8 (mmol/L), 6.9 ± 0.9 (mg/L), respectively. Patients in the control groups had SCR, BUN, and Hs-CRP factors of 265.9 ± 77.3 (mol/L), 16.3 ± 1.9 (mmol/L), and 5.9 ± 0.9 (mg/L), respectively, following therapy, as shown in Table 4.

**TABLE 4:** Improvement of function indexes before and after treatment.

Groups	n	Time	SCR(mol/L)	BUN(mmol/L)	Hs-CRP(mg/L)
Treatment groups	40	Before treatment	364.3 ± 56.9	50.2 ± 3.2	8.5 ± 1.7
		After treatment	301 ± 47.3	38.1 ± 2.8	6.9 ± 0.9
Control groups	40	Before treatment	366.9 ± 41.9	39.8 ± 2.9	6.9 ± 1.6
		After treatment	265.9 ± 77.3	16.3 ± 1.9	5.9 ± 0.9

## 5 Factors affecting the prognosis of SARF

### 5.1 Etiology

In recent years, studies have found that severe acute renal failure has multiple causes, sometimes not a single factor but a combination of multiple factors. High incidence of cardiovascular and gastrointestinal diseases have mortality. Severe acute renal failure with secondary obstructive disease and urological surgery is associated with lower mortality. Therefore, it is necessary to clarify the pathogenesis of renal failure and clarify the nature of renal failure.

### 5.2 Severity of disease before treatment

The conservative treatment groups and the renal replacement therapy groups in this paper have little difference in the treatment effect. Specifically, this paper analyzes its reasons. The renal function status of dialysis patients is better than that of conservative treatment before treatment, but the effect of dialysis treatment varies depending on the etiology, which often aggravates in the initial stage, and the complications and complications increase, such as concurrent multiple organ dysfunction syndrome, cardiovascular disease, and diabetes, thus causing the patient to enter into a dangerous period. In this study, disease severity is associated with prognosis before treatment. Especially in cardiovascular disease, respiratory failure, sepsis and liver dysfunction, the survival rate decreased significantly.

### 5.3 Age

Numerous studies have reported an association between age and survival. However, due to the poor compensatory ability of the elderly and underlying cardiovascular diseases such as diabetes, hypertension, coronary heart disease, etc., elderly patients are prone to complications such as severe infection, metabolic acidosis, and hyperkalemia. Age is found to be a prognostic factor in this study, but treatment should not be dismissed just because of age. For the treatment of acute renal failure in the elderly, it is usually necessary to choose symptomatic treatment according to the cause of the disease and the patient’s condition, and correct the patient’s water electrolyte and acid-base balance. During the treatment period, the patient’s underlying diseases and risk factors should be paid attention to and the overall prevention and treatment work should be actively carried out to improve the clinical treatment effect and reduce the mortality rate.

### 5.4 Treatment methods

Active treatment of the primary disease and early selection of appropriate renal replacement therapy are the key points to improve the cure rate. Severe acute renal failure is a severe disease characterized by multiple organ failure, hemodynamic instability, large release of inflammatory mediators, and severe immunosuppression. Continuous blood purification can greatly improve the above symptoms. Severe acute renal failure is usually treated with continuous renal replacement therapy. In this paper, the method of combining blood perfusion and hemodialysis (IHD) was selected. Combination therapy with hemodialysis is a treatment method for poisoned patients (paraquat poisoning). Hemodialysis is suitable for removing those that are water soluble and not bound to proteins and other components in plasma. However, previous studies have confirmed that hemoperfusion is significantly higher than hemodialysis for the clearance of high fat-soluble and protein-binding substances. Therefore, blood perfusion is generally preferred for acute renal failure caused by severe drug and poison poisoning. Because it has a stronger ability to remove personal substances or proteins,” has been added to the text to further supplement it. In addition, there are also special cases such as acute kidney injury caused by some poisoning, and acute kidney injury caused by acute drug poisoning on the basis of the original chronic kidney disease. At this time, hemoperfusion combined with hemodialysis should be used. The treatment method can remove water and uremic toxins, correct electrolyte and acid-base disorders, and achieve the removal of toxic substances. Different treatment methods of renal replacement therapy are of great significance to the continuous and slow elimination of metabolic waste and excess water in the body, the comprehensive correction of electrolyte and acid-base disorders, and the stability of the internal environment. Of course, in the course of drug treatment, renal replacement therapy should not be ignored. Aggressive management of the underlying disease, early detection of disease-causing risk factors, and prevention of other complications are required. Early identification of disease risk factors is also important to prevent other complications.

### 5.5 Reasonable nutrition

The data of this groups showed that the mortality rate of the normal groups was 27.5%, which was higher than that of the observation groups, which was 17.5%. Reasonable nutrition means that the body can take in to maintain good health. This suggests that reasonable nutritional support is helpful to improve prognosis and reduce morbidity and mortality. In addition, the change table of clinical indicators before and after treatment showed that albumin decreased after treatment. The aggravation of nutritional status in the short term is mainly caused by the following factors. Postoperative wound aggravation or infection leads to a large loss of nutrients. Insufficient nutrient intake is characterized by anorexia, nausea, vomiting, hemorrhage, and other causes of azotemia and metabolic disturbances caused by impaired glucose utilization and protein synthesis. Continuous hemodialysis is an important treatment, but it also affects nutritional support because some nutrients are lost through semipermeable membranes.

Currently, replacement therapy (CRRT) and hemodialysis (IHD) are of great significance for the treatment of severe acute renal failure. It can not only remove excess water in the body and correct acidosis and electrolyte imbalance, but also prevent and reduce the occurrence of complications such as multiple organ dysfunction syndrome to a certain extent, which improves patient survival. There are intermittent hemodialysis and continuous renal replacement therapy. Alternative therapy has incomparable special effects on a variety of acute and critical diseases. Substitution therapy removes metabolites and toxins in a slow, continuous and isotonic manner to maintain stable internal environment and stable hemodynamics, thereby creating favorable conditions for nutritional support for acute and critically ill patients with severe acute renal failure. Therefore, early, continuous and adequate renal replacement therapy is very necessary in patients with severe acute renal failure.

The results of this study show that elderly patients often suffer from decreased renal function or multi-organ disease, which makes them a high-risk area for severe acute renal failure. Rapid decline of renal function will lead to edema, urine increase, low back pain and other symptoms, which will seriously affect the life safety of patients. After therapy, patients in the experimental groups’s endogenous blood creatinine rate was noticeably greater than that of patients in the control groups, and their blood urea nitrogen and creatinine levels were lower than those of the control groups. It shows that hemodialysis treatment can effectively improve the glomerular filtration rate and renal function, which is beneficial to the recovery of body function. Conservative treatment mostly uses diuretics and volume expanders dopamine and furosemide. However, the efficacy is limited and the clinical symptoms are not significantly improved. In contrast, hemodialysis treatment helps to remove excess water and metabolic waste by expelling human blood from the body and exchanging substances using the principles of diffusion, adsorption, ultrafiltration and convection. It helps to maintain a stable internal environment, effectively restore kidney function, reduce blood urea nitrogen and creatinine content, and increase endogenous creatinine clearance.

Through the analysis of the causes of patients, the characteristics and principles of prevention and treatment of such diseases can be more deeply understood. The occurrence of most severe acute renal failure diseases has been truncated from the source to reduce the probability of their occurrence, which has improved the cure rate of severe acute renal failure by 9.47%.

## 6 Conclusion

In this study, individuals with severe acute renal failure who had hemodialysis (IHD) and replacement treatment (CRRT) were examined. Clinical data revealed that the observation groups’s parathyroid hormone and renin levels were much higher than the control groups, and that observation groups’s calcium and phosphorus metabolism was significantly better than that of the control groups. In conclusion, hemodialysis has a significant effect on patients with severe acute renal failure, which can effectively improve the content of blood urea nitrogen and creatinine and increase the survival rate. In patients, continuous renal replacement treatment can efficiently lower parathyroid hormone and renin levels compared to intermittent hemodialysis. The calcium and phosphorus metabolism has been controlled, and the quality of life has been improved.

## Data Availability

The original contributions presented in the study are included in the article/Supplementary Material, further inquiries can be directed to the corresponding author.

## References

[B1] Ayad S. Elkattawy S. Ejikeme C. Al-nasseri A. Reddy A. (2021). Acute renal failure in a patient with severe acute respiratory syndrome coronavirus 2. Cureus 478 (6), 331–354. 10.7759/cureus.13406 PMC7983180 33767927

[B2] Bagshaw S. M. Wald R. (2017). Strategies for the optimal timing to start renal replacement therapy in critically ill patients with acute kidney injury. Kidney Int. 91 (5), 1022–1032. 10.1016/j.kint.2016.09.053 28222898

[B3] Block G. A. Bushinsky D. A. Cunningham J. Drueke T. B. Ketteler M. Kewalramani R. (2017). Effect of etelcalcetide vs placebo on serum parathyroid hormone in patients receiving hemodialysis with secondary hyperparathyroidism: Two randomized clinical trials. Jama 317 (2), 146–155. 10.1001/jama.2016.19456 28097355

[B4] Castillo-Torres S. A. Ibarra-Sifuentes H. R. Sánchez-Terán H. Sánchez-Martínez C. Chávez-Luévanos B. Estrada-Bellmann I. (2018). Restless legs syndrome in end-stage renal disease patients undergoing hemodialysis. Arq. Neuro-Psiquiatria 76 (12), 827–830. 10.1590/0004-282X20180133 30698206

[B5] Chen T. M. Chen Y. P. (2019). Comparative study of continuous renal replacement therapy and intermittent hemodialysis in severe acute renal failure. J. Gannan Med. Univ. 229 (6), 228–261.

[B6] Cikova A. Vavrincova-Yaghi D. Vavrinec P. Dobisova A. Gebhardtova A. Flassikova Z. (2017). Gastrointestinal tuberculosis following renal transplantation accompanied with septic shock and acute respiratory distress syndrome: A survival case presentation. Bmc Gastroenterol. 17 (1), 131–137. 10.1186/s12876-017-0695-5 29179699 PMC5704353

[B7] Delmas C. Zapetskaia T. Conil J. M. Georges B. Vardon-Bounes F. Seguin T. (2018). 3-month prognostic impact of severe acute renal failure under veno-venous ECMO support: Importance of time of onset. J. Crit. Care 44 (9), 63–71. 10.1016/j.jcrc.2017.10.022 29073534

[B8] Hawkins A. M. Jesuthasan L. Vardesh D. L. (2018). Case of severe acute lupus myocarditis and multiple-organ failure. Bmj Case Rep. 201 (5), bcr2018225085. 10.1136/bcr-2018-225085 PMC6011492 29925558

[B9] Kirsch A. H. Lyko R. Nilsson L. G. Amdahl M. Lechner P. (2017). Performance of hemodialysis with novel medium cut-off dialyzers. Nephrol. Dial. 32 (1), 165–172. 10.1093/ndt/gfw310 PMC5837492 27587605

[B10] Kistler B. M. Benner D. Burrowes J. D. Campbell K. L. Fouque D. Garibotto G., (2018). Eating during hemodialysis treatment: AConsensus statement from the international society of renal nutrition and metabolism. J. Ren. Nutr. 28 (1), 4–12. 10.1053/j.jrn.2017.10.003 29249295

[B11] Lin S. Y. Yi-Wen C. (2018). Three months of rifapentine and isoniazid for latent tuberculosis infection in hemodialysis patients: High rates of adverseevents. J. Microbiol. Immunol. Infect. 168 (4), 118–127. 10.1016/j.jmii.2018.05.003 29907535

[B12] Lischkova L. (2018). Severe suicidal self-poisoning with massive dose of potassium ferricyanide: Risk of life-threating hyperkalemia and acute renal failure. Clin. Toxicol. 56 (6), 538–539.

[B13] Lumlertgul N. Peerapornratana S. Trakarnvanich T. Pongsittisak W. Surasit K. Chuasuwan A. (2018). Early versus standard initiation of renal replacement therapy in furosemide stress test non-responsive acute kidney injury patients (the FST trial). Crit. Care 22 (1), 101–118. 10.1186/s13054-018-2021-1 29673370 PMC5909278

[B14] Lv Z. Qiao L. (2020). Analysis of healthcare big data. Future Gener. Comput. Syst. 109, 103–110. 10.1016/j.future.2020.03.039

[B15] Robinski M. Mau W. Wienke A. Girndt M. (2017). The choice of renal replacement therapy (CORETH) project: Dialysis patients' psychosocial characteristics and treatment satisfaction. Nephrol. Dial. Transplant. 339 (4), 315–324. 10.1093/ndt/gfv464 28186578

[B16] Sabatino A. Regolisti G. Karupaiah T. Sahathevan S. Sadu Singh B. K. Khor B. H. (2017). Protein-energy wasting and nutritional supplementation in patients with end-stage renal disease on hemodialysis. Clin. Nutr. 229 (3), 663–671. 10.1016/j.clnu.2016.06.007 27371993

[B17] Shafi T. Powe N. R. Meyer T. W. Hwang S. Hai X. Melamed M. L. (2017). Trimethylamine N-oxide and cardiovascular events in hemodialysis patients. J. Am. Soc. Nephrol. 28 (1), 321–331. 10.1681/ASN.2016030374 27436853 PMC5198291

[B18] Srinivasa K. G. Sowmya B. J. Shikhar A. Utkarsha R. Singh A. (2018). Data analytics assisted internet of things towards building intelligent healthcare monitoring systems: Iot for healthcare. J. Organ. End User Comput. 30 (4), 83–103. 10.4018/joeuc.2018100106

[B19] Wang C. Lv L. S. Huang H. Guan J. Ye Z. Li S. (2017). Initiation time of renal replacement therapy on patients with acute kidney injury: A systematic review and meta‐analysis of 8179 participants. Nephrology 326 (1), 7–18. 10.1111/nep.12890 27505178

[B20] Xie H. Chen S. (2018). Effects of continuous renal replacement therapy and intermittent hemodialysis on severe acute renal failure. Chin. Foreign Med. Res. 314 (5), 155–174.

[B21] Xie S. Yu Z. Lv Z. (2021). Multi-disease prediction based on deep learning: A survey. Comput. Model. Eng. Sci. 127 (3), 489–522. 10.32604/cmes.2021.016728

[B22] Yang X. M. Tu G. W. Zheng J. L. Shen B. Ma G. G. Hao G. W. (2017). A comparison of early versus late initiation of renal replacement therapy for acute kidney injury in critically ill patients: An updated systematic review and meta-analysis of randomized controlled trials. Bmc Nephrol. 18 (1), 264–277. 10.1186/s12882-017-0667-6 28784106 PMC5547509

[B23] Zin K. Akula M. (2021). Abstract: Delayed rebound hypercalcemia in severe rhabdomyolysis-induced acute renal failure. Endocr. Pract. 27 (6), 261–274. 10.1016/j.eprac.2021.04.663 33588062

